# Marijuana Use in Aesthetic Surgery Patients: A Retrospective Review of 441 Cases

**DOI:** 10.1093/asjof/ojae007.025

**Published:** 2024-04-12

**Authors:** Veronique Doucet, Avinash Islur

**Affiliations:** University of Manitoba, Winnipeg, MB, Canada; University of Manitoba, Winnipeg, MB, Canada

## Abstract

**Goals/Purpose:**

Marijuana use is increasing in Canada following its legalization in 2018. 27% of Canadians were reported to have consumed marijuana in 2022. Marijuana use in surgical patients is a topic that has had exponential growth in the literature recently. The drug has many therapeutic effects such as analgesia, muscle relaxation, sedation and mood improvement. However, it is also associated with deleterious cardiovascular, respiratory and coagulopathic effects that can significantly impact the care of surgical patients in the peri-operative period. Literature from other surgical specialities has shown similar recovery and ultimate surgical outcome between marijuana users and non-marijuana users despite increased pain and poorer quality of life associated with marijuana use. There is a paucity of information about the effects of marijuana on aesthetic plastic surgery outcomes. The prevalence of marijuana use in aesthetic plastic surgery patients is currently unknown and there is a need for more evidence to develop clinical practice guidelines regarding the use of marijuana in the perioperative period. The purpose of this study is to describe the effects of marijuana consumption on aesthetic plastic surgery outcomes.

**Methods/Technique:**

A single-center retrospective review was completed including all patients who underwent abdominoplasty, mastopexy and/or other body contouring surgery (such as brachioplasty, thigh lift or lower body lift) between January 2021 and August 2023. Other procedures such as liposuction, fat grafting, implant insertion or removal were also reviewed if they took place during the same general anesthetic. Marijuana use was defined as use within 4 weeks pre- and/or post-operatively. Data collection included patient demographics, body mass index (BMI), marijuana use, smoking status, comorbidities, surgical procedure(s) performed, operative time, resection weight and/or liposuction volume (if applicable), complications and follow up.

**Results/Complications:**

A total of 1000 procedures in 441 patients were reviewed during the 32-month study period. Average patient age was 43 years old and average patient BMI was 27.3 kg/m^2^. 20.4% of patients were marijuana users. The average number of procedures completed per patient was 2.3 and average operative time was 167 minutes (2 hours and 47 minutes). 79% of cases involved more than one surgical procedure. 63% of the 441 cases involved an abdominoplasty, 49% involved a mastopexy, 5% involved a brachioplasty and 4% involved a thigh lift procedure. 33% of cases included breast implants and 47% liposuction. Average follow up time was 5.2 months. Overall surgical complications consisted of a 5% superficial infection rate, 1% deep infection rate, 9% seroma rate, 1% hematoma and lymphocele rates, 3% rate of wound dehiscence requiring surgical intervention and 11% rate of superficial delayed wound healing. There were no cases of nipple necrosis or full thickness skin necrosis. A comparison of patient demographics between marijuana and non-marijuana user groups revealed that marijuana users were significantly younger than non-marijuana users (average age 39 and 44 years old respectively). Surgical data, outcomes and complications were not found to be significantly different between the 2 groups for all variables.

**Conclusion:**

Marijuana consumption in the perioperative period does not appear to affect aesthetic surgery outcomes such as post-operative infections, seroma, lymphocele, wound dehiscence, or fat necrosis based on the results of this study. There is a need for prospective work on this topic to produce better quality evidence and ultimately to create evidence-based guidelines on the perioperative use of marijuana for aesthetic surgery patients.

